# A scoping review of home-produced heroin and amphetamine-type stimulant substitutes: implications for prevention, treatment, and policy

**DOI:** 10.1186/s12954-016-0105-2

**Published:** 2016-04-19

**Authors:** Evelyn Hearne, Jean-Paul Cornelius Grund, Marie Claire Van Hout, Jim McVeigh

**Affiliations:** School of Health Sciences, Waterford Institute of Technology, Waterford, Ireland; Department of Addictology, 1st Faculty of Medicine, Charles University, Prague, Czech Republic; CVO – Addiction Research Centre, Utrecht, The Netherlands; Freudenthal Institute for Science and Mathematics Education, Utrecht University, Utrecht, The Netherlands; Centre for Public Health, Liverpool John Moores University, Liverpool, UK

**Keywords:** Homemade drugs, Scoping studies, Krokodil, Internet, Kitchen chemistry

## Abstract

Several home-produced substances such as *krokodil* and *boltushka* are prevalent in many Eastern European countries. Anecdotal reports of its use have been circulating in Germany and Norway; however, this has not been confirmed. Its use has also been reported by the media in the USA, although only one confirmed report of its use exists. Home-produced drugs are associated with high levels of morbidity and a number of complex health issues such as the spread of *blood borne viruses*, *gangrene*, and *internal organ damage*. The high incidence of HIV rates amongst people who inject home-produced substances is a public health concern. The resulting physical health consequences of injecting these crude substances are very severe in comparison to heroin or amphetamine acquired in black markets. Due to this fact and the increased mortality associated with these substances, professionals in the area of prevention, treatment, and policy development need to be cognisant of the presentation, harms, and the dangers associated with home-produced substances globally. This scoping review aimed to examine existing literature on the subject of home-produced heroin and amphetamine-type stimulant substitutes. The review discussed the many implications such research may have in the areas of policy and practice. Data were gathered through the use of qualitative secondary resources such as journal articles, reports, reviews, case studies, and media reports. The home production of these substances relies on the utilisation of precursor drugs such as less potent stimulants, tranquillizers, analgesics, and sedatives or natural plant ingredients. The Internet underpins the facilitation of this practice as recipes, and diverted pharmaceutical sales are available widely online, and currently, ease of access to the Internet is evident worldwide. This review highlights the necessity of prevention, education, and also harm reduction related to home-produced drugs and also recommends consistent monitoring of online drug fora, online drug marketplaces, and unregulated pharmacies.

## Background

The use of drugs in countries of the former socialist republic is not a recent phenomenon [1, 2]. Following the Former Soviet Union’s (fSU) downfall in December 1991, with the addition of massive social and economic collapse in Eastern Europe, came the escalating problem of illicit drug use [1–4] especially in Russia, the Ukraine, the Baltic States, and most other former Soviet republics [1, 2, 5–10]. Indeed, homemade drug cultures emerged already in the fSU in the late 1970s and 1980s, as well as in, Czechoslovakia, Poland, and Hungary [1, 11, 12]. Monetary restrictions and closed borders that preluded the Soviet Union’s downfall prohibited individuals from acquiring the substances emerging in the counter cultures of Western Europe and the USA [1, 2]. As a result, all the countries emerging from the Soviet Union have a shared history of widespread homemade drug use, primarily opiates and amphetamine-type stimulants (ATS), through the usage of natural elements, diverted pharmaceutical drugs, and even household chemicals [4, 13, 14].

A foremost concern for public health and drug policy is the diversion and misuse of pharmaceuticals [15]. The Internet underpins this concern as both recipes of drug chemistry and diverted pharmaceuticals are available online and the Internet is now accessible to virtually anyone worldwide [16]. United Nations Office on Drugs and Crime (UNODC) reported an increase in the diversion of pharmaceuticals for non-medicinal use in many countries including Nigeria, the USA, Hong Kong, Sweden, Australia, Canada, Indonesia, Germany, and China [16, 17].

There are a substantial number of physical harms resulting from injecting homemade drug solutions and countless dangers connected to the practice of home cooking of heroin and ATS substitutes such as the spread of blood borne viruses (BBVs), skin and soft tissue infections, and even chemical injuries and burns as a result of explosions during the cooking process [14, 18–23]. Cooks and consumers alike are exposed to these chemicals, as are potentially their families and the environment [24, 25]. Further research in this area will be of great benefit to healthcare workers, treatment providers, and policy makers. As the consequences of injecting these homemade substances are considerably more acute than existing illicit narcotics [26], and life expectancy lower [19], treatment providers globally should be cognisant of the dangers of, presentation, and harms related to homemade drug use. Policy makers should be responsive, as homemade drug use in countries outside of Eastern Europe may be probable for reasons such as the current global economic climate and the effects of resulting austerity measures on vulnerable communities such as heroin users in Greece turning to cheap homemade methamphetamine; mephedrone and MDPV taking over people who inject drugs (PWID) scenes in Romania; and effortless access to unregulated pharmacies and online drug markets [16]. By scoping the literature, healthcare workers and treatment providers will benefit, and highlighted gaps in current research should inform practice and policymakers. An incidence of this type of drug production may be detrimental to PWID and become a major public health concern. This scoping study will focus on the history of homemade drug use worldwide but particularly in Eastern Europe, as it is more prevalent there. The harms associated with the practice of producing and consuming homemade drugs will be highlighted for harm reduction purposes and aimed toward prevention, treatment, and policy.

### The scoping review

**Fig. 1:**
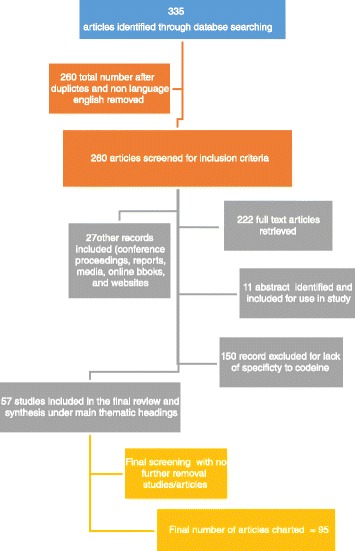
Flow diagram charting inclusion and exclusion criteria for this study

Scoping studies are gradually being encouraged for the extensive searching of literature on specific subjects. They are primarily used to emphasise the gaps and key issues in the current evidence base and to find areas that require further research, practical, and policy interventions [15, 27–29]. It is important to acknowledge the limitations of a scoping study. As the quantity of data generated in a scoping study is sometimes considerable, the decision to include all material available versus a more detailed analysis of a smaller number of studies can be difficult. Scoping studies do not appraise the evidence quality in the primary research papers, and as a result, scoping studies simply offer a descriptive or narrative interpretation of available research [16, 27]. This scoping review employed qualitative secondary sources together with peer reviewed journal articles, reports, reviews, case studies, and some media accounts. A thorough list using many different search terms was used to perform a literature search. These terms included “*homemade drugs*”, “*kitchen chemistry*”, “*krokodil*”, “*desomorphine*”, “*boltushka*”, “*drug formulation tampering*”, *and* “*online drug markets*”. To guarantee all articles relevant to the study were included, a broad search was conducted using many databases: EBSCO Host, Science Direct, PubMed, PsycINFO, and MEDLINE. A set of criteria for inclusion and exclusion in the study were put in place. Inclusion criteria consisted of home-produced substances limited to ATS and heroin substitutes and full-text access. Exclusion criteria consisted of incomprehensible language, animal studies, and insignificance to the scoping review (see Fig. 1 and Table 1).

**Table 1:** Categories used to organise the literature

Homemade drug use in Eastern Europe	
Homemade drug use outside of Eastern Europe	
Heroin and amphetamine-type stimulants/substitutes	
Harms associated with homemade drugs	
Drug and formulation tampering	
The Internet	
Scoping studies	

### Heroin and amphetamine-type stimulants

Heroin addiction is defined as a chronic relapsing condition that, for many, is an unrelenting, lifelong illness with severe effects. This is particularly relative to short life expectancies and high rates of morbidity [30–33]. Ninety percent of the world’s heroin supply is directly from opium grown in Afghanistan. Heroin that is produced from Afghan poppies is shipped worldwide [34]. With an estimated 3.1 million consumers, Europe is the main destination for Afghan heroin. In Europe, the Russian Federation is by far the largest consumer of Afghan heroin [34] with 2.3 % of its population injecting opioid drugs, indicative of Russia’s proximity to Afghanistan [23]. Approximately 1.5 million people consume heroin in the Russian Federation and 1.6 million dispersed over other European countries [34].

The most widespread used opiate worldwide is Codeine, an alkaloid prepared from opium via a process known as methylation [35–37]. Codeine is employed in several different ways such as a sedative, analgesics, treatment of tuberculosis, and anti-diarrhoeal [36, 38]. In recent years, codeine-containing cough syrups (CCS) have been seen as independent substances of abuse and moreover as substitution for conventional drugs of abuse, e.g. amphetamines, cocaine, and opiates [37, 39, 40]. Combination analgesics containing codeine (CACCs) are a mixture of codeine and other substances, e.g. ibuprofen, paracetamol, or aspirin. CACCs are obtainable over-the-counter (OTC) drugs in some countries worldwide such as Ireland (Solpadeine®), Australia (Panadeine®), and the Ukraine (Codelac® and Terpincod®). These sales of CACCs were banned in Russia in June 2012 [22]; however, codeine has simply moved to the black market and is therefore still available. CACCs bought over the counter are considered “*safe*” to treat pain, if used in accordance with the recommended dosages. Yet, persistent long-term use and unnecessary dosing patterns can result in dependence physically and psychologically; continual headaches as a result of overuse of the medication; and a myriad of other complex medical issues [41, 42].

Amphetamine-type stimulants (ATS) include amphetamine, d-amphetamine, methamphetamine, methylphenidate, 3,4-methylenedioxymethamphetamine (MDMA) and also cathinone, methcathinone, pseudoephedrine, fenetylline, and ephedrine [43–45]. These are a sizeable collection of psychoactive compounds that all contain natural elements in their chemical structure [45]. ATS operate on a person’s nervous system and have powerful effects on the individual’s mind and body. Some of these include appetite suppression, heart rate elevation, intense happiness, and mental and energy awareness. Although ATS are characteristically controlled substances, a number of them are regulated and utilised for treating disorders such as attention deficit hyperactivity disorder (ADHD), narcolepsy, and depression that is resistant to treatment [46]. There currently stands a general non-medicinal use of such stimulants [45, 47] with ATS production and manufacture occurring in numerous parts of the world [48]. Indeed, ATS use is more prevalent than cocaine or opiates [49]. It is suggested that there are 35 million people using ATS worldwide, compared with 29 million people consuming opiate and/or cocaine [34, 48, 50]. There are several variations in the form of ATS produced globally, e.g. in Europe, ATS manufacture is primarily in tablet or powder forms of ecstasy (MDMA) and amphetamine. In the Czech Republic and other countries of Central and Eastern Europe, homemade methamphetamine is locally known as “*Pervitin*”, “*Vint*”, *or* “*Shirka*” [1, 2, 51].

### History of homemade drug use

Throughout the 1970s, information on Western youth and counter culture increasingly triggered the interest in psychoactive drugs amongst young adolescents. The practice of home-cooked drugs most likely commenced 10 to 15 years before the political changes, on the fringes of dissident intellectual “*third culture*” or “*underground*” circles. The growing aversion of the stifling Soviet ideology made these young people equally distrustful and disdainful of the harsh Soviet anti-drug propaganda—often phrased in the anti-Western *Agit-Prop* discourse and images they were subjected to. Keen on emulating the experiences of their western peers, Soviet Youth took their parents’ tradition of Samogon to the next level [1, 2] and used precursor drugs from natural plant ingredients or over-the-counter or prescription drugs with psychoactive properties—containing less potent stimulants, tranquillizers, analgesics, and sedatives—to produce their own drugs [1, 10, 14, 19, 20, 52].

In Poland, students from the University of Gdansk (the birthplace of the Solidarity movement) reportedly first synthesised “*kompot*,” homemade heroin, by extracting the opioids from poppy straw and acetylation of the morphine content [11, 53]. In early 1980s Prague, “*Freud*” had taken a few chemistry courses at a technical university before he first synthesised the (in)famous “*pervitin*” or “*piko*” (methamphetamine) from ephedrine, the active ingredient in over-the-counter cold medications [54]. Both drugs rapidly diffused amongst young people in both countries in the 1990s. Kompot was the driving force behind the 1980s HIV epidemic amongst PWID in Poland [5]. Although the Czech Republic has been spared widespread HIV infection amongst PWID, pervitin remains the problem child on the Czech drug scene [1, 54, 55].

During the 1980s these recipes diffused across most Soviet countries, but much less in its Central European vassal states. With that came adjustments of formulae and chemicals used in the synthesis [44], leading to a simpler, cruder process and a range of monikers. Heroin made from poppies became known under the moniker of “*cheornaya*” (black, referring to the colour of the final liquid drug) and “*hanka*” in Russia and “*shirka*” in Ukraine.

Homemade methamphetamine became “*vint*”^1^ in Russian and methcathinone “*jeff*”, or “*mulka*”, while in Ukrainian, these drugs were termed “*belyi*”^2^ and “*ephedrone*”, while cathinone is termed “*Bolthuska*”^3^ [1, 44]. This argot is often very local and remarkably elastic. In some Russian towns, methamphetamine was called belyi/beloye and vint in many places in Ukraine. The word belyi is also used for ephedrine or methcathinone or may refer to any stimulants (white for stimulants; black for opiates).

Both the home-produced opioids and ATS are produced using caustic chemicals, such as sulphuric acid (H2SO4), phosphorus, iodine, and industrial or household solvents by (often unskilled) cooks under the most primitive laboratory conditions—in kitchens and basements. It should not come as a surprise that much of these corrosive reactants remain present in the final drug [44]. The grave and harsh reality of this practice is that the substances finally produced come primarily in liquid form, ready for injection, which is the usual mode of use in the region [7].

**Fig. 2:**
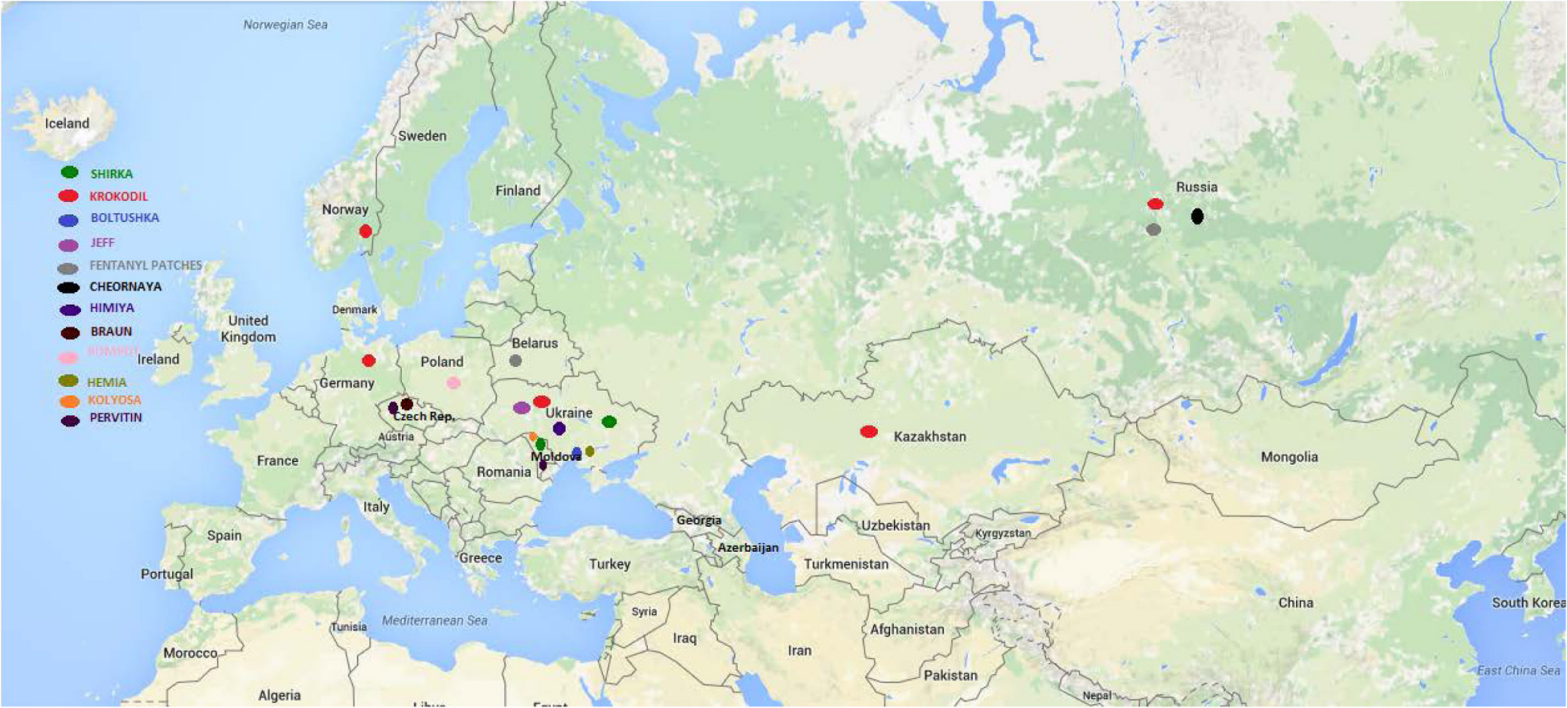
Summary map

The phenomenon of homemade drug use continues to influence Eastern European drug culture (see Fig. 2) [53]. Early 2011 saw a remarkable escalation in the number of reports in the media of the use of a new homemade drug known as “*krokodil*” (Russian: крокодил), also “*Russian Magic*”, “*crocodile*”, or by its chemical name “*Desomorphine*” [19, 20]. Research has shown that this home-produced opiate first appeared in Russia around 2002/2003 [19, 20, 23], although Czech drug cooks also modestly produced a drug from codeine based analgesics in the 1980s, known as “*braun*” or “*brown*” due to its colour [1, 2] (see Table 2) [16].

**Table 2:** Homemade drug solutions, street names, ingredients, geographic area of use and key public health concerns

Street name	Drug type/chemical	Ingredients	Key adverse health and social consequences	Country/city of use
Krokodil. Other common street names: Russian Magic, Crocodile, Russian Heroin	Opiate-desomorphine	Codeine, gasoline or paint thinner, iodine, red phosphorous, tropicamide	Injecting risks for BBV transmission and risks present in the production process, contamination, chemical reaction, sharing of paraphernalia, and group injecting practices. High mortality rates. Krokodil users presenting in surgeries/emergency rooms with serious and advanced medical complications. Undesirable medical and social costs.	Russia, Ukraine, Georgia, Kazakhstan. Germany (Bochum, Berlin, Frankfurt), Norway (Tromsø). Also: Anecdotal reports in UK, Czech Republic, France, Belgium
Boltushka. Other common street names: Baltushka, Balka	Cathinone	Ephedrine, pseudoephedrine, warm water, household vinegar, and potassium permanganate	Injecting risks for BBV transmission and risks present in the production process, contamination, chemical reaction, sharing of paraphernalia, and group injecting practices. Long-term users of *boltushka* can experience partial loss of cognitive function, Alzheimer-type symptoms, and possible brain damage. Manganese-induced Parkinsonism, which is irreversible. Undesirable medical and social costs.	Odessa*
Jeff Other common street names: Jaff, Cat, Mulka, Ephedrone	Methcathinone	Phenylpropanolamine, warm water, household vinegar, and potassium permanganate	Injecting risks for BBV transmission and risks present in the production process, contamination, chemical reaction, sharing of paraphernalia, and group injecting practices. Manganese-induced Parkinsonism. Injecting risks for BBV transmission. Undesirable medical and social costs.	Ukraine
Fentanyl patches (new and used). Other common street names: China White, White Persian	Opiate	Fentanyl, acetaminophen, caffeine	Fentanyl use is associated with increased odds of overdose. Criminality such as misusers who resort to obtaining used patches from elderly nursing home residents, and searching hospital and nursing home dumpsters for discarded patches. Undesirable medical and social costs.	Russia, Belarus
Cheornaya	Opiate	Poppy Straw, cigarette ash, sodium bicarbonate	Access to poppy straw is seasonal and when it is scarce, injectors will turn to other opiate-type drugs. Injecting risks for BBV transmission and risks present in the production process, contamination, chemical reaction, sharing of paraphernalia, and group injecting practices. Circulatory damage and soft tissue infections amongst injectors. Undesirable medical and social costs.	Russia
Himiya	Opiate	Poppy straw	Access to poppy straw is seasonal and when it is scarce, injectors will turn to other opiate-type drugs, including krokodil. Injecting risks for BBV transmission and risks present in group injecting practices. Undesirable medical and social costs.	Ukraine
Braun Other common street name: Brown	Opiate	Mixture of morphine and codeine products, e.g. hydrocodone	Injecting risks for BBV transmission and risks present in the production process, contamination, sharing of paraphernalia, and group injecting practices.	Czech Republic
Kompot Other common street names: Polish heroin	Opiate	Poppy straw, acetic anhydride, acetone	Access to poppy straw is seasonal and when it is scarce, injectors will turn to other opiate-type drugs. Injecting risks for BBV transmission Guillain-Barré Syndrome. Injecting risks for BBV transmission and risks present in group injecting practices. Undesirable medical and social costs.	Poland
Shirka (Ukraine) Other common street names: Cherniashka, Black, Hanka	Opiate	Poppy straw	Access to poppy straw is seasonal and when it is scarce, injectors will turn to other opiate-type drugs Injecting risks for BBV transmission and risks present in group injecting practices. Undesirable medical and social costs.	Ukraine, Moldova
Shirka (Odessa*)	Methamphetamine	Ephedrin, Pseudoephedrine	Binge-using patterns that enhance the probability of unintentional overdoses. Injecting risks for BBV transmission, i.e. risks present in the production process, contamination, sharing of paraphernalia, and group injecting practices. Undesirable medical and social costs.	Odessa*
Hemia	Opiate	Poppy straw	Access to poppy straw is seasonal and when it is scarce, injectors will turn to other opiate-type drugs, including krokodil and kolyosa. Injecting risks for BBV transmission and risks present in group injecting practices. Undesirable medical and social costs.	Odessa*
Kolyosa	Opiate	Mixture of codeine-containing pills	Users will turn to this due to poppy straw being unavailable with associated overdose and injecting risks. Injecting risks for BBV transmission and risks present in the production process, contamination, sharing of paraphernalia, and group injecting practices. Undesirable medical and social costs.	Moldova
Pervitin Other common street name: Vint, Piko	Methamphetamine	Ephedrine, pseudoephedrine, industrial chemicals such as gasoline, toluene and tetrachlorethylene	Binge-using patterns that enhance the probability of unintentional overdoses. Injecting risks for BBV transmission and risks present in the production process, contamination, sharing of paraphernalia, and group injecting practices. Undesirable medical and social costs.	Czech Republic, Moldova

^*^ Ukraine - refers to all cities in Ukraine, except Odessa, which has a range of different terms/names for their homemade drugs

Desomorphine’s sedative properties are ten times stronger than the effects of heroin. However, it has a shorter half-life; therefore, dependence may rapidly appear with continued administration [19, 20]. There are both serious short- and long-term negative health consequences and high mortality rates associated with krokodil, primarily resulting from the crude desomorphine extraction and the failure to separate or filter out its numerous toxic by-products [19, 20, 56–58]. Evidence of widespread use of an injectable home-produced cathinone known as “*boltushka*” was noted by Chintalova-Dallas et al. [44]. This ATS was initially reported in Odessa, Ukraine in 2005. Boltushka is produced by mixing vinegar, warm water, potassium permanganate (KMnO4) together with the precursor, phenylpropanolamine (PPA) from crushed *“koldack”* or *“teffedrin”* tablets [44, 59–61]. The tradition of home-produced drugs has been reported in several other countries such as Norway [14, 62, 63], Georgia, Ukraine, Kazakhstan [14, 23, 64], New Zealand [65], and Germany [14, 63, 66] where the use of krokodil is reported [16]. Greece reported a homemade version of methamphetamine called “*shisa*” or “*drug of the poor*” [67, 68], while in the Netherlands and other European Countries, the use of homemade gamma-hydroxybutyric acid (GHB) is a small but intensive problem [69, 70]. Reports indicate that the occurrence of homemade drug use has transpired as a result of reduced heroin availability in Russia, the Baltic States, and the five central Asian countries [16, 22, 55, 71–73].

### Drug and formulation tampering and the Internet

Using illicit drugs has now been exceeded by the non-medicinal use and abuse of diverted pharmaceuticals in the USA [13, 74]. (Pseudo) Ephedrine, codeine, codeine cough syrups (CCS), and fentanyl patches are the most ordinarily tampered with pharmaceuticals. Lankenau et al. [75] suggest that formulation tampering with these drugs allows for dispensing higher doses and therefore is cost effective. Motivations for tampering with pharmaceuticals involve numerous reasons including increasing the bio-availability of the drug, quicker onset of the effects, and to boost the drugs psychoactive effects [16]. Sedatives, stimulants, analgesics, and tranquillizers are broadly pursued, measured, and tampered with for the purpose of recreational intoxication [13, 76]. The Internet is accessible to many people worldwide and is a prime source of information for recreational drug consumers, interested in tampering with formulations due to the wealth of information on websites, including drug user fora, providing potential home cooks with advice and tips on the techniques and recipes. This is a concern for public health, given the pervasive nature of the Internet worldwide [13, 16]. Previous successes as well as failures are documented and discussed (see www.erowid.org; www.bluelight.ru; www.drugs-forum.com) [13, 77]. Advice includes description of methods for tampering such as crushing, separating, purifying, and optimum usage [13, 78–83]. Moreover, online market places found on the “*deep web*” or “*darknet*” are now accessible. The deep web is described as the section of the Internet that is not searchable with established search engines, e.g. google. The darknet is described as a small area within the deep web and has been purposefully concealed and cannot be accessed via usual web browsers [84]. The most well-known online market is the Silk Road Marketplace which was active between Feb 2011 and October 2013, and was followed by Silk Road 2.0 active from November 2013 to November 2014 [85]. Although Silk Road and Silk Road 2.0 have been shut down, other online marketplaces can be found on the “deep web” where the site owners, buyers, and vendors can stay somewhat anonymous due to their IP addresses being masked and random routing via peer-based computer networks, using the TOR browser [85–87]. These online marketplaces are innovative new avenues for drug sales [88, 89, 90, 91], facilitating anonymous acquisition and supply of licit drugs, pharmaceuticals, and illicit drugs alike [91]. Additionally, the Internet hosts a number of drug discussion fora, wherein users publicly and anonymously exchange knowledge. Several Darknet drug markets have discussion fora as well, where, for example, the quality of the drugs and vendors are rated and discussed. These drug fora offer users practical tools prior to purchase and use of substances and indigenous harm reduction. Numerous studies have emphasised the importance of harm reduction occurring amongst these online communities [92–96]. Between 2010 and 2012, Russian policy makers highlighted the negative effect the Internet had in the dissemination of information related to krokodil production and use. A marked rise in online searches for krokodil preparation and methods to purchase was noted [16]. However, a major reduction in Internet searches for krokodil related information was noted after the ban on pharmaceutical sales of codeine, June 1, 2012 [22].

### Harms associated with homemade drug use: implications for harm reduction

The well-established tradition of “*kitchen chemistry*” [16] is still dominating stimulant and opioid use in many parts of Eastern and Central Europe [23]. These homemade drugs are rife in marginalised strata of society such as amongst people with lower socioeconomic status or homeless individuals, due to their low costs and widespread availability of OTC or diverted pharmaceuticals and easy access to recipes [16, 23, 97]. In Russia, the process for producing krokodil only comprises of a small amount of precursor pharmaceuticals, e.g. one to five packets of codeine based analgesics or 80 to 400 mg of codeine and takes approximately 45 min to cook, sometimes less. Although other home-produced substances are not without harms, krokodil seems to be associated with particularly severe complications and ghastly health outcomes [10]. The severe morbidity associated with injecting krokodil is likely a function of the short half-life of the drug, dictating frequent injection, missing or inapt reactants, and incomplete synthesis, leaving large fractions of reactants and resulting in an extremely corrosive drug cocktail [14, 65].

The transmission of BBVs is a major health concern associated with any form of injecting drug use [16, 22]. The primary causes of BBV transmission are the collective use of injecting equipment (needle sharing) and sharing of liquefied drugs [98–101]. These behaviours are also important drivers of skin and soft tissue infections (SSTIs) around injection sites, affecting 10–30 % of PWID [102–104] and associated with loss of venous access and intramuscular or subcutaneous injection [102, 104, 105]. It has been documented that there is a high risk of BBV transmission amongst PWID, through the sharing not only of needles but also of other equipment used in the process. HIV and hepatitis C infection rates amongst PWID are extremely high in Russia, Ukraine, Georgia, and other fSU countries [106]. These first two countries are reported as the most affected by krokodil use [23].

Grund et al. [14] summarised the major concerns related to krokodil use, as reported in the literature and by PWID, such as skin irritations, ulcers, scale-like skin deformations that eventually turn green (like a crocodile skin) and black (necrotic), jaw osteonecrosis, thrombophlebitis, muscle damage, thyroid injury, inflammation of kidneys and the liver, endocrine complications, amputation of limbs and, ultimately, death [16, 18–20, 56, 107, 108]. Reportedly, limb amputations or jaw removals are often the only lifesaving intervention [20, 57, 108]. Reportedly, people who inject krokodil often present at medical services in the later stages of disease because they fear medical stigma and close ties between medical providers and law enforcement or other systems of social control, such as child protection agencies [14, 109–111].

Krokodil is not the only homemade substance to have severe physical complications. In recent years, several Eastern European countries have reported “*Manganese Induced Parkinsonism*” associated with injection of “*boltushka*” (homemade cathinone). First described as “*Manganism*”, as early as 1837 [112], overexposure to manganese is a severe condition that can become manifest after only a few months of boltushka injecting, with symptoms of dysarthria, hypokinesia, dystonia, and damaged posture [113–115]. Boltushka synthesis includes the oxidation of (the precursor) with permanganate or “marganzovka”, a commonly used disinfectant in Russia, in water [44]. During the reaction, Manganese (Mn) is released and toxic levels of remnants remain in the liquid drug. The rapid progression of Manganism amongst boltushka consumers [115, 116] points at short term, continuous exposure to perhaps extremely high concentrations of manganese. Although data are missing, we suggests that manganese concentrations in people who inject homemade (meth)cathinone may exceed levels amongst people affected by industrial pollution and perhaps even those of workers in the battery manufacturing and manganese processing industries, who, reportedly, are most at risk [117]. This is a serious concern for PWID, treatment providers, healthcare workers, and policy makers alike as the resulting Parkinsonism syndrome is not reversible [44]. Studies suggest Manganism related to (meth)cathinone injection amongst immigrants in Western Europe and in Canada [116]. Individuals have reported experiencing manganese toxicity from using MCAT (4-methylmethcathinone) [118] in online drug community forums [119].

Countries outside of Eastern Europe should be well-informed about these grave public health concerns. A variety of opioid and stimulant syntheses are described in detail on the Internet, and the precursors and reactants are readily available. This may lead to such harmful substances emerging in unexpected settings. Indeed, traditional drug diffusion theory [120, 121] accounts poorly for the way in which new drug trends spread geographically, from one locality to another, and culturally, between different social groups or communities, and also how information about their risks is being shared. Emerging drug trends no longer necessarily commence in (cultural) capitals, harbour cities or along (physical) drug trading routes. Indeed, in the *glocalised* and *Vernetzte* twenty first century, *iDrugs* and new drug trends may emerge in any municipality, large or small, urban, or rural. Digitalisation of drug markets, immigration, and global travel may also be of significant influence [16].

Due to the adverse health issues associated with these homemade substances, governmental reaction is necessary so as to increase the regulation of over the counter and prescribed medications, and also to provide coordinated services such as counselling services, medical supports, wound and infection management, testing and support for HIV, outreach, and rehabilitation services for users of the substances [16, 20, 58, 65, 122–126]. Development of harm reduction tactics such as hygiene education, needle exchanges, bleach distribution, provision of filters, foil packs to try encourage users to reverse route of administration, provision of safer recipes for home-produced substances, treatment such as opiate substitution and antiretroviral therapy, and prevention programmes are vital [16, 44, 123–126]. Continuing research that will explore users’ awareness of harms of homemade drugs, user practices, users’ experiences of services, and trajectories of use are fundamental to informing harm reduction approaches [14, 16]. Continued surveillance and monitoring at harm reduction programmes using internal data systems to monitor new trends is warranted.

## Conclusion

Home-produced substances that replace illicit ones, such as heroin and amphetamines, are associated with many complex health issues and high levels of morbidity. This scoping review has presented extant literature on the topic and highlights how this issue is a growing and concerning public health imperative warranting drug user and online surveillance, targeted harm reduction and clinical responses.

## Abbreviations

ADHD: attention deficit hyperactivity disorder
ATS: amphetamine-type stimulant
BBV: blood borne virus
CACC: combination analgesics containing codeine
CCS: codeine-containing cough syrup
fSU: Former Soviet Union
GHB: gamma-hydroxybutyric acid
H2SO4: sulphuric acid
KMnO4: potassium permanganate
MCAT: 4-methylmethcathinone (4-MMC)
MDMA: 3,4-methylenedioxymethamphetamine
MDPV: methylenedioxypyrovalerone
OTC: over the counter
PWID: people who inject drugs
SSTIs: skin and soft tissue infections
UNODC: United Nations Office on Drugs and Crime

## Footnotes

1 “Screw,” with reference to some of its less pleasurable side-effects.

2 “White”.

3 “Chatterbox”.
